# High-Resolution Genotyping of the Endemic *Salmonella* Typhi Population during a Vi (Typhoid) Vaccination Trial in Kolkata

**DOI:** 10.1371/journal.pntd.0001490

**Published:** 2012-01-31

**Authors:** Kathryn E. Holt, Shanta Dutta, Byomkesh Manna, Sujit K. Bhattacharya, Barnali Bhaduri, Derek J. Pickard, R. Leon Ochiai, Mohammad Ali, John D. Clemens, Gordon Dougan

**Affiliations:** 1 Department of Microbiology and Immunology, University of Melbourne, Melbourne, Australia; 2 National Institute of Cholera and Enteric Diseases, Kolkata, India; 3 Wellcome Trust Sanger Institute, Hinxton, Cambridge, United Kingdom; 4 International Vaccine Institute, Seoul, Korea; University of California San Diego School of Medicine, United States of America

## Abstract

**Background:**

Typhoid fever, caused by *Salmonella enterica* serovar Typhi (*S.* Typhi), is a major health problem especially in developing countries. Vaccines against typhoid are commonly used by travelers but less so by residents of endemic areas.

**Methodology:**

We used single nucleotide polymorphism (SNP) typing to investigate the population structure of 372 *S.* Typhi isolated during a typhoid disease burden study and Vi vaccine trial in Kolkata, India. Approximately sixty thousand people were enrolled for fever surveillance for 19 months prior to, and 24 months following, Vi vaccination of one third of the study population (May 2003–December 2006, vaccinations given December 2004).

**Principal Findings:**

A diverse *S.* Typhi population was detected, including 21 haplotypes. The most common were of the H58 haplogroup (69%), which included all multidrug resistant isolates (defined as resistance to chloramphenicol, ampicillin and co-trimoxazole). Quinolone resistance was particularly high among H58-G isolates (97% Nalidixic acid resistant, 30% with reduced susceptibility to ciprofloxacin). Multiple typhoid fever episodes were detected in 22 households, however household clustering was not associated with specific *S.* Typhi haplotypes.

**Conclusions:**

Typhoid fever in Kolkata is caused by a diverse population of *S.* Typhi, however H58 haplotypes dominate and are associated with multidrug and quinolone resistance. Vi vaccination did not obviously impact on the haplotype population structure of the *S.* Typhi circulating during the study period.

## Author Summary

Typhoid fever is caused by the bacterium *Salmonella enterica* serovar Typhi (*S.* Typhi) and is a major health problem especially in developing countries. Vaccines against typhoid are commonly used by travelers but less so by residents of endemic areas. We used single nucleotide polymorphism (SNP) typing to investigate the population structure of 372 *S.* Typhi bacteria isolated from typhoid patients during a typhoid disease burden study and Vi anti-typhoid vaccine trial in Kolkata, India. Approximately sixty thousand people were enrolled for fever surveillance for 19 months prior to, and 24 months following, vaccination of one third of the study population against typhoid (May 2003–December 2006, vaccinations given December 2004). We detected a diverse population of *S.* Typhi, including 21 different genetic forms (haplotypes) of the bacteria. The most common (69%) were of a haplogroup known as H58, which included all multidrug resistant isolates (bacteria resistant to the antibiotics chloramphenicol, ampicillin and co-trimoxazole). Resistance to quinolones, a class of antibiotics commonly used to treat typhoid fever, was particularly high among a subgroup of H58 (H58-G). Vi vaccination did not obviously impact on the haplotype distribution of the *S.* Typhi circulating during the study period.

## Introduction

Salmonella *enterica* serovar Typhi (*S.* Typhi) is the bacterium responsible for typhoid fever, which affects more than 20 million people each year, resulting in over 200,000 deaths [1], [2]. As *S.* Typhi is transmitted by the fecal-oral route, the typhoid fever burden falls almost exclusively in developing areas where sanitation is poor [1], [3]. The current mainstay of typhoid fever treatment is antimicrobial therapy [4], however resistance to antimicrobials is common among *S.* Typhi [5], leading to prolonged bacterial clearance times and treatment failure [6], [7]. Children and young adults are the most vulnerable population for developing typhoid fever [1], [8], [9] and can be protected by vaccination against *S.* Typhi [10], [11]. However while vaccines against *S.* Typhi are frequently used by travelers to typhoid endemic areas [12], they are yet to be effectively harnessed for the protection of local, typhoid endemic populations [13].

*S.* Typhi is a highly clonal bacterium estimated to have entered the human population on a single occasion approximately 50,000 years ago [14]. We have recently identified hundreds of single nucleotide polymorphisms (SNPs) within the *S.* Typhi chromosome that are suitable for rapidly and informatively subtyping *S.* Typhi populations [15], [16]. As recombination is rare in *S.* Typhi, SNP typing allows individual *S.* Typhi isolates to be assigned unequivocally to unique haplotypes. Importantly, as haplotypes are defined by phylogenetically informative sequence variation, SNP typing also reveals information about genome sequence and the evolutionary relationship between isolates [15], [16]. As our SNP panel is designed to allow inference of phylogenetic relationships, it does not target SNPs that are likely to be under selection, such as drug resistance loci. SNP haplotyping studies in localized areas where typhoid is endemic, including Jakarta [17], Kathmandu [18], [19], the Mekong Delta [20] and Nairobi [21], have revealed that the typhoid burden in endemic areas is usually attributable to a diverse population of differentiable *S.* Typhi haplotypes, co-circulating within the local human population. These studies also revealed the clonal expansion of a *S.* Typhi haplogroup, H58, in South East Asia [16], [18], [20], as well as in Nairobi [21].

During 2003–2004, a typhoid burden study was conducted in a typhoid endemic area of Kolkata, India [8], [22], [23]. This was followed by a large-scale, cluster-randomized phase IV trial to determine the efficacy of the injectable Vi polysaccharide vaccine (Typherix, GlaxoSmithKline) among the local population (>60,000 persons). The study site was divided into 80 geographic clusters (40 clusters each randomly assigned to Vi vaccine or inactivated hepatitis A vaccine as a control) and in December 2004, eligible residents were vaccinated (mean 60% of the population vaccinated in each cluster) [11]. The primary results of the trial, namely 61% efficacy among vaccinees and indirect protection within and around Vi vaccinated geographic clusters, have been published elsewhere [11], [24]. Surveillance for fever was conducted uninterrupted throughout May 2003–December 2006, and typhoid fever was confirmed by positive blood culture of *S.* Typhi [8], [11], [22], [23]. A total of 372 typhoid cases were confirmed by blood culture during the study period, including 197 during the post-vaccination period. All *S.* Typhi isolates produced Vi during *in vitro* culture [11], however Vi expression is tightly regulated in *S.* Typhi growing on laboratory media and *in vivo* [25], [26], [27] and we consequently hypothesised that selection against Vi expression in Vi immunized individuals might result in differential efficacy of Vi vaccine against different *S.* Typhi phylogenetic lineages. Here we present an analysis of the 372 *S.* Typhi isolates collected during the study period, including SNP haplotyping, antimicrobial susceptibility profiling, analysis of intra-household transmission and determination of Vi vaccine efficacy for the most common circulating haplotypes.

## Methods

### Bacterial isolates

A total of 372 *S.* Typhi were isolated during the typhoid disease burden study from May 2003 to December 2006, intervened by a Vi effectiveness trial (December 2004), conducted in Kolkata, India [8], [11], [22], [23]. *S.* Typhi were isolated from blood cultures of fever patients following standard techniques [28]. The institutional review boards at the International Vaccine Institute, the National Institute of Cholera and Enteric Diseases, and the Indian Council of Medical Research approved the protocol and monitored the progress of the studies. All subjects provided written informed consent for vaccination and oral informed consent for blood culture (for children, informed consent was provided by their guardian). The assayed isolates represent all confirmed typhoid cases during the study period May 2003 to December 2006, among subjects who were present in the field area at baseline, including 10 cases in non-vaccinees that were not included in the original vaccine report due to incomplete demographic data [11]. Confirmation of *S.* Typhi was done by agglutination with poly and monovalent antisera (BD diagnostics, US), Vi phenotype was checked by agglutination with monovalent Vi antisera.

### Antimicrobial susceptibility testing

Testing was performed using Kirby Baure's disc diffusion method using 11 antimicrobial discs from BD diagnostics (ampicillin, tetracycline, chloramphenicol, cotrimoxazole, nalidixic acid, ciprofloxacin, ofloxacin, ceftriaxone, amikacin, aztreonam, amoxicillin-clavulanic acid). MICs of antimicrobials were determined by E-test (AB Biodisk, Solna, Sweden) and interpreted following CLSI guidelines [29]. Multidrug resistance (MDR) was defined as simultaneous resistance to chloramphenicol (MIC>256 µg/mL), ampicillin (MIC>256 µg/mL) and co-trimoxazole (MIC>32 µg/mL).

### DNA extraction and SNP typing

DNA extraction was carried out from overnight LB culture of *S.* Typhi isolates using Promega DNA extraction kit following manufacturer's instructions. DNA samples were quantified using the Quant-IT kit (Qiagen, USA) and concentrations adjusted to 10 ng/µl using nuclease-free water (Ambicon, USA). SNP typing was performed using either GoldenGate or Sequenom assays (loci in Table S1). The former was performed using a GoldenGate custom array according to the manufacturer's standard protocols (Illumina, USA), covering 1,500 loci (Table S1) as described previously [18], [20], [21]. Briefly, DNA samples were arrayed in a 96-well plate along with a negative control (water) and positive control (sequenced Typhi), assayed using two custom oligo pools (200 SNPs included on both arrays for quality control) using the Illumina GoldenGate platform and analyzed using Illuminus-P [21]. Sequenom assays of 100 loci (Table S1) were performed using the iPLEX Gold assay (Sequenom Inc, USA), designed using the MassARRAY Assay Design software version 3.1 (Sequenom Inc, USA) as previously described [19]. Samples were amplified in multiplexed PCR reactions before allele specific extension. Allelic discrimination was obtained by analysis with a MassARRAY Analyzer Compact mass spectrometer. Genotypes were automatically assigned and manually confirmed using MassArray TyperAnalyzer software version 4.0 (Sequenom Inc, USA).

### Phylogenetic and statistical analysis

**Figure 1:**
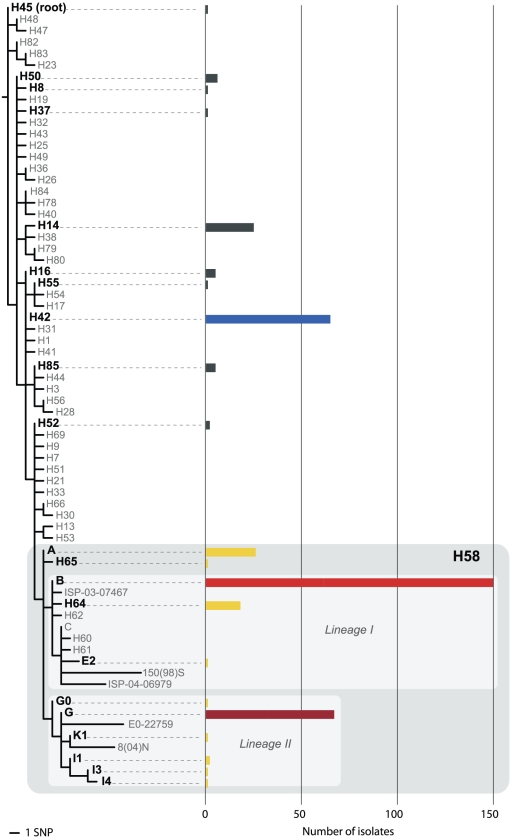
*S.* Typhi haplotypes identified by SNP typing. Rooted phylogenetic tree indicating *S.* Typhi haplotypes defined by assayed SNPs, scale as indicated. Haplotypes identified among 372 Kolkata isolates are labeled in black, the number of detected isolates for each haplotype is indicated by bars according to the scale at the bottom. Bars are coloured to indicate major haplotypes, as in Figures 2, 3. The H58 haplogroup is highlighted in grey, and is further divided into two major lineages I and II as indicated.

Phylogenetic analysis (Figure 1) was based on 81 SNPs common to both GoldenGate and Sequenom assays (Table S1), which include those dividing isolates into 48 major haplotypes (original defined in [16]) and further subdivision of the H58 haplogroup into subtypes (originally defined in [15]). Each isolate was assigned to a node in the previously defined *S.* Typhi phylogenetic tree based on alleles at these 81 SNP loci. Statistical analysis was performed in *R* [30]. Haplotype-specific typhoid isolation rates in Vi vaccinees vs hepatitis A vaccinees (Table 1) were compared using Fisher's exact test (two-tailed test).

**Table 1:** Efficacy of Vi vaccine against major *S.* Typhi haplotype groups following vaccination.

	Vaccinees				Non-Vaccinees			
	Hep A (typhoid cases)	Vi (typhoid cases)	Rate ratio	VE [95% CI]	Hep A (typhoid cases)	Vi (typhoid cases)	Rate ratio	VE [95% CI]
**H58**	65	28	0.43	57% [33, 72]**	24	15	0.66	34% [−26, 65]
**H42**	19	4	0.21	79% [38, 93]*	7	2	0.70	30% [−45, 94]
**Other**	12	2	0.17	83% [26, 96]*	5	4	0.84	16% [−214, 77]
**Total**	96	34	0.35	65% [48, 76]**	36	21	0.62	38% [−5, 64]
Persons	18,804	18,869			12,877	12,206		

*S.* Typhi isolated during January 2005–December 2006 (i.e. within 24 months post-vaccination). Vi, geographical clusters randomly assigned to Vi vaccine against *S.* Typhi; Hep A, control clusters assigned to hepatitis A vaccine; VE, vaccine efficacy;

*p<0.01;

**p<0.001 (Fisher's exact test). There was no evidence for difference in efficacy between haplotypes (p>0.3, Fisher's exact test).

## Results

### Population structure of *S.* Typhi in Kolkata

All 372 *S.* Typhi isolates collected between May 2003 and December 2006 were subjected to SNP haplotyping using high-throughput Sequenom or Illumina GoldenGate platforms (Table S1). These two genotyping methods have been applied previously to study *S.* Typhi populations [17], [18], [19], [20]. Forty-five of the assayed loci were discovered by mutation analysis of 200 gene fragments within a global collection of *S.* Typhi [16] and provide medium-level resolution of the *S.* Typhi population, subdividing it into 48 distinct haplotypes (displayed as a phylogenetic tree in Figure 1). Eleven of these haplotypes, which are broadly distributed across the tree, were identified among the Kolkata *S.* Typhi (Figure 1, excluding shaded area). The globally dominant haplotype H58 was by far the most common (N = 260, 70%), followed by H42 (N = 65, 17%) and H14 (N = 25, 7%) (Figure 1). We assayed 50 additional SNP loci, discovered by whole genome sequence analysis of seven globally distributed *S.* Typhi H58 isolates [15], that provide greater resolution within the H58 haplogroup and subdivide it into 20 distinct subtypes (Figure 1, shaded area). Eleven H58 subtypes were identified among the Kolkata *S.* Typhi (Figure 1), however 97% of H58 isolates belonged to just four H58 subtypes: B (N = 148, 40% of all *S.* Typhi tested), G (N = 66, 18%), A (N = 22, 6%) and H64 (N = 17, 5%).

### *S.* Typhi haplotypes associated with antimicrobial resistance

Resistance to the quinolone Nalidixic acid (Nal) was common (54% of all isolates), with Nal resistance observed among phylogenetically unlinked haplotypes (Table 2), indicating that Nal resistance arises frequently within distinct *S.* Typhi chromosomal backgrounds. Each common haplotype included isolates that were Nal resistant but susceptible to ciprofloxacin, as well as isolates that were Nal resistant and exhibiting reduced susceptibility to ciprofloxacin (MIC≥0.125 µg/mL) (Table 2). Interestingly the most common haplotype, H58-B, exhibited low rates of Nal resistance, with only 24% of H58-B isolates displaying resistance to Nal (significantly lower than other H58 (94% resistant), p<10^−8^ using Fisher's exact test). The highest rate of Nal resistance was observed among the second-most common haplotype, H58-G, with 97% of isolates resistant to Nal and 31% also exhibiting reduced susceptibility to ciprofloxacin (Table 2). Two isolates were ciprofloxacin resistant (MIC≥16 µg/mL) and have been described in detail elsewhere [31]. These isolates were of identical haplotype, H58-I1 (see Figure 1) and isolated from siblings (aged 3 and 5 years) on the same day in July 2004 [31]. No other isolates of this haplotype were detected during the study (2003–2006). Multiple drug resistance (MDR, defined as resistance to chloramphenicol, ampicillin and co-trimoxazole) was observed in 43 *S.* Typhi isolates (11.5%), of which most (N = 38) were also Nal resistant. The MDR *S.* Typhi isolates belonged to five H58 subtypes: H58-A (5 isolates), H58-B (12 isolates), H58-G (10 isolates), H64 (a sub-type of H58) (15 isolates) and H58-I4 (1 isolate). These subtypes occupy the internal nodes of the H58 phylogeny, including members of both major lineages (see Figure 1), indicating that MDR is widely distributed among the H58 haplogroup.

**Table 2:** Distribution of quinolone resistance phenotypes among *S.* Typhi haplotypes.

Haplotype	Nal^R^				Nal^S^	Total
	Cip^S^	Cip^I^	Cip^R^	All		
**H58 subtypes**						
**- A**	14	3	0	17	5	22
**- B**	23	13	0	36	112	148
**- E2**	1	0	0	1	0	1
**- G**	43	20	0	63	2	65
**- G0**	1	0	0	1	0	1
**- H64**	12	5	0	17	0	17
**- H65**	1	0	0	1	0	1
**- I1**	0	0	2*	2	0	2
**- I3**	1	0	0	1	0	1
**- I4**	1	0	0	1	0	1
**- K1**	1	0	0	1	0	1
**All H58**	98	41	2*	141	119	260
**H14**	14	5	0	19	6	25
**H16**	1	0	0	1	4	5
**H42**	24	6	0	30	35	65
**H50**	4	0	0	4	2	6
**H85**	5	0	0	5	0	5
**Other**	1	0	0	1	5	6
**Total**	147	52	2*	201	171	372

Haplotypes are defined in Figure 1. Nal^S^: Nalidixic acid susceptible (MIC<8 µg/mL); Nal^R^: Nalidixic acid resistant (MIC>256 µg/mL); Cip^S^: ciprofloxacin susceptible (MIC<0.125 µg/mL); Cip^I^: ciprofloxacin reduced susceptible (MIC≥0.125 µg/mL); Cip^R^: ciprofloxacin resistant (MIC>1 µg/mL);

*Cip MIC>16 µg/mL.

### Temporal patterns and the effect of vaccination on the *S.* Typhi population

**Figure 2:**
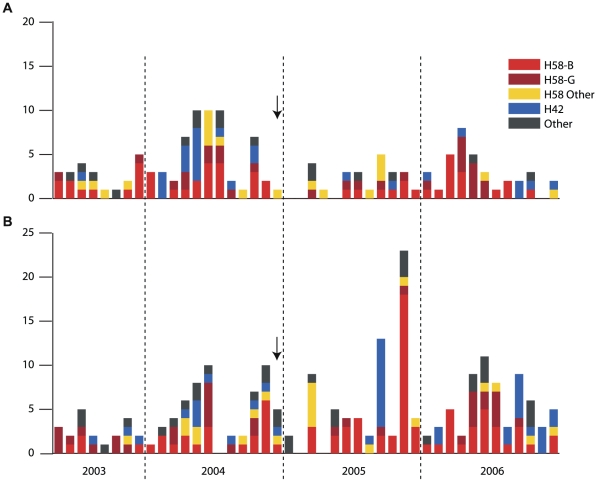
Temporal distribution of *S.* Typhi haplotypes. Monthly frequency of *S.* Typhi coloured by haplotype (haplotypes defined in Figure 1). Vaccines were administered in December 2004 (indicated by arrows) to approximately two thirds of the study population. (A) *S.* Typhi isolated from typhoid fever patients in geographical clusters assigned to Vi vaccine. (B) *S.* Typhi isolated from typhoid fever patients in geographical clusters assigned to hepatitis A vaccine.

The incidence of typhoid fever remained high throughout the four-year study period, with a median of seven cases per month and no clear seasonal pattern (Figure 2). A total of 168 *S.* Typhi were isolated during May 2003–November 2004 (19 month pre-vaccination period), 7 during December 2004 (vaccination period) and 197 during January 2005–December 2006 (24 month post-vaccination period). The same haplotypes dominated throughout the study (Figure 2), indicating that the burden of typhoid fever in Kolkata was the result of a diverse range of co-circulating haplotypes. One exception to this pattern was a peak in typhoid cases in November 2005 involving 27 infections, of which 21 were *S.* Typhi H58-B consistent with a small outbreak (Figure 2), although no spatial clustering was evident. Only 2 of the 21 H58-B cases in this month occurred in clusters assigned to Vi vaccine, suggesting the vaccine was effective in providing protection during the outbreak (Figure 2).

**Figure 3:**
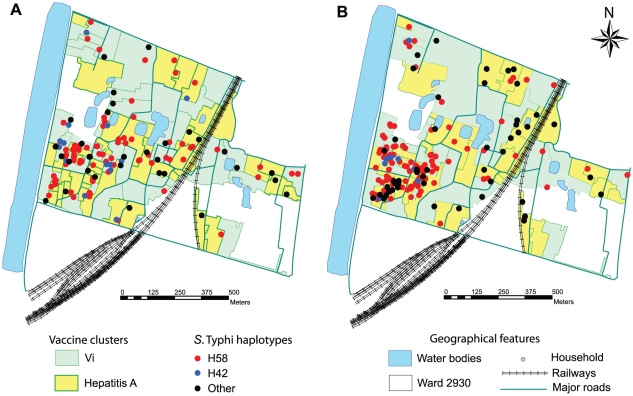
Spatial distribution of *S.* Typhi haplotypes. Maps of the study site, illustrating the division of the site into 80 geographical clusters randomly assigned to receive Vi or control (hepatitis A) vaccine. The location of each typhoid fever patient's residence is indicated by a star, coloured by the haplogroup of the corresponding *S.* Typhi isolate. (A) *S.* Typhi isolated before administration of vaccine (May 2003–November 2004). (B) *S.* Typhi isolated after administration of vaccine (January 2005–December 2006).

As previously reported, the incidence of typhoid fever during the two years following vaccination was >60% lower among individuals who received the Vi typhoid vaccine than those who received hepatitis A vaccine (Table 1) [11]. Our haplotype data indicates this overall reduction was due to a statistically significant reduction in isolation rate across all *S.* Typhi haplotypes (H58, H42 and others, see Table 1). All *S.* Typhi isolated during the study, including those from individuals who had been vaccinated with Vi (‘breakthrough cases’), reacted strongly with commercially available Vi antisera (BD diagnostics, USA) in an agglutination test, indicating that all strains could express the vaccine target Vi. The 34 *S.* Typhi isolates from breakthrough cases belonged to several distinct SNP haplotypes and were also diverse in terms of antimicrobial resistance (Table 3).

**Table 3:** Haplotypes and antimicrobial sensitivity phenotypes for *S.* Typhi isolated from individuals vaccinated with Vi.

Haplotype	Resistance phenotype	No. isolates
H14	Nal^R^	1
H37	S	1
H42	S	1
H42	Nal^R^	3
H58-A	Nal^R^	1
H58-B	S	11
H58-B	Nal^R^	3
H58-G	MDR	1
H58-G	Nal^R^	7
H58-G	Nal^R^, MDR	3
H58-H64	Nal^R^	1
H58-H64	Nal^R^, MDR	1

Haplotypes correspond to those defined in Figure 1. Nal^R^: Nalidixic acid resistant (MIC>256 µg/mL); MDR: multidrug resistant, defined as resistant to chloramphenicol (MIC>256 µg/mL), ampicillin (MIC>256 µg/mL) and co-trimoxazole (MIC>32 µg/mL); S: susceptible to all antimicrobials tested.

### *S.* Typhi haplotypes from households with multiple typhoid infections

There were 22 households from which multiple *S.* Typhi were isolated by blood culture (21 households with 2 positive cultures; 1 household with 3 positive cultures, total 45 positive cultures; Table 4). For three of these households, the paired isolates resulted from two blood cultures from the same individual, taken 3–5 weeks apart and thus representing possible cases of relapse or re-infection. Each of these isolate pairs displayed identical *S.* Typhi haplotypes and resistance phenotypes, consistent with relapse as opposed to re-infection with a distinct haplotype (Table 4). However different *S.* Typhi haplotypes were involved in each pair of these relapse cases, and displayed different antimicrobial resistance profiles (H14, Nal^R^; H58-G, MDR; H64, Nal^R^+MDR).

**Table 4:** Details of households with multiple confirmed typhoid fever cases.

		Case 1		Case 2		Time to 2^nd^ case	Different haplotype	Relapse/reinfection
House	Cluster	Hap	Vi vacc	Hap	Vi vacc			
**A**	Hep A	H14	-	H14	-	17 days	-	yes
**B**	Hep A	H64	-	H64	-	23 days	-	yes
**C**	Vi	H58-G	yes	H58-G	yes	34 days	-	yes
**D**	Hep A	H58-G	-	H58-G	-	0 days	-	-
**E**	Vi	H42	-	H42	-	8 days	-	-
**F**	Hep A	H42	-	H42	-	8 days	-	-
**G**	Hep A	H14	-	H14	-	8 days	-	-
**H**	Hep A	H58-B	-	H58-B	-	10 days	-	-
**I**	Hep A	H58-B	-	H58-B	-	22 days	-	-
**J**	Hep A	H58-G	-	H58-G	-	28 days	-	-
**K**	Hep A	H58-B	-	H58-B	-	34 days	-	-
**L**	Hep A	H58-B	-	H58-B	-	2 mo	-	-
**M**	Hep A	H58-B	-	H58-B	-	2 mo	-	-
**N**	Hep A	H58-B	-	H58-B	-	>10 mo	-	-
**O**	Hep A	H58-B	-	H58-B	-	>1 yr	-	-
**P**	Vi	H85	-	H64	-	33 days	yes	-
**Q**	Hep A	H58-B	-	H58-G	-	2 mo	yes	-
**R**	Hep A	H58-G	-	H14	-	>3 mo	yes	-
**S**	Hep A	H58-B	-	H58-G	-	>4 mo	yes	-
**T**	Hep A	H58-B	-	H58-A	-	>4 mo	yes	-
**U**	Hep A	H55	-	H58-G	-	>1 yr	yes	-
**M** *	Hep A	H58-B	-	H42	-	1 yr	yes	-
**V**	Vi	H42	-	H58-B	yes	>2 yr	yes	-

House = household identifier; Cluster = geographic cluster for vaccine trial; Hap = *S.* Typhi haplotype; Vi vacc = received Vi vaccination prior to typhoid fever episode; Case 1, Case 2 = first and second case occurring in household; Time to 2nd case = time between *S.* Typhi-positive blood culture collection from cases 1 and 2; Different haplotype = case 2 haplotype differs from first case; Relapse/reinfection = case 2 occurred in same individual as case 1;

*three cases detected in household, row indicates comparison of case 1 to case 3.

In the remaining 19 households with multiple cases, *S.* Typhi was isolated from different individuals, thus representing distinct typhoid cases within the same household. Among these, twelve households had more than one typhoid case occurring within two months (Table 4). In nearly all of these households, the same *S.* Typhi haplotype (displaying same resistance phenotype) was isolated from both cases, consistent with direct transmission between household members or a shared environmental source such as food or water (10/12 households, Table 4, p = 0.039 using Binomial test with equal probability of same or different haplotypes). Among households in which a second typhoid case occurred more than two months after the first, the later infection was most often caused by a distinct *S.* Typhi haplotype (5/7 households, Table 4). To examine whether Vi vaccination reduced intra-household transmission, we compared the proportion of cases for which apparent transmission was observed in the same household (defined as the same *S.* Typhi haplotype isolated from another member of the household one week to two months after the initial case), among Hepatitis A and Vi clusters in the post-vaccination period. While it is possible that two typhoid cases caused by the same haplotype in the same household could result from shared exposure to a common source of *S.* Typhi, it is more likely that infections separated by more than a week constitute transmission events. Using this definition of household transmission, six percent (8/135) of cases in the Hepatitis A cluster were linked to putative transmission within a household, while none of the 59 cases in the Vi cluster were obviously linked to transmission. While the numbers are low, this provides weak evidence for protection against person-to-person or direct transmission by the Vi vaccine (p = 0.045 using Fisher's exact test), which may be via direct protection of vaccinated individuals and/or indirect protection via herd immunity in clusters assigned to the Vi vaccine. The distribution of haplotypes among these likely transmission events was no different to that of haplotypes among all Hepatitis A clusters during the post-vaccination period (5 cases, 8% for H58-B; 1 case, 6% for H580-G; 1 case, 4% for H42; p = 1 using Fisher's exact test). Thus there is no evidence that any particular haplotype is more likely to be transmitted person-to-person.

## Discussion

### *S.* Typhi populations in Kolkata and other typhoid endemic areas

SNP typing of *S.* Typhi isolated during 2003–2006 revealed a diverse range of haplotypes co-circulating in the study site, an urban slum area in eastern Kolkata. A similar level of diversity has been observed in previous studies in typhoid endemic areas [17], . The dominant *S.* Typhi haplotypes were subtypes of H58, collectively accounting for 70% of all *S.* Typhi isolated during the four-year study (Figure 1). The dominance of H58 has been reported in recent studies of *S.* Typhi infections in other typhoid endemic areas including Kathmandu, Nepal (69% H58, 2003–2004) [18], the Mekong Delta, Vietnam (98% H58, 2004–2005) [20] and Nairobi, Kenya (87% H58, 2001–2008) [21]. However there does appear to be greater diversity within the H58 group in Kolkata. We identified 11 distinct H58 haplotypes, including four with high frequency among *S.* Typhi from Kolkata (18–150 isolates each) including the ancestral node (A) and nodes from both major lineages of H58 (Figure 1). In neighbouring Nepal, two hospital-based studies of S. Typhi found 61–69% of isolates belonged to a single subtype of H58 lineage II, H58-G, and few other H58 isolates were detected [18], [19]. In the Mekong Delta, Vietnam, a large hospital-based study found 95% of *S.* Typhi isolated from adults and children with typhoid fever belonged to one of three closely related H58 lineage I subtypes, H58-C, -E1 and -E2 (see Figure 1) [20]. In that study, differentiation of the three subtypes was possible because the genome of an isolate from the study had been sequenced for the purpose of SNP discovery [15].

### Diversification and differentiation within *S.* Typhi H58

The H58 subtypes that were common in the present study in Kolkata are internal nodes of the H58 phylogenetic tree described by the assayed SNPs (A, B, G, H64, see Figure 1). This is not particularly surprising, since SNP discovery for our assays did not include analysis of any Kolkata strains, with the exception of two isolates of the H64 haplotype (actually part of the H58 group, see Figure 1) which were included in mutation detection within 200 gene fragments [16] but not at a genome-wide scale [15]. Since SNPs accumulate locally over time as bacteria replicate, we would expect that there is more diversity in the Kolkata *S.* Typhi population than we are able to detect in our SNP assays (i.e. mutations have occurred locally at genomic positions that we did not assay). If more Kolkata isolates had been included in SNP discovery, we would be able to differentiate among Kolkata isolates at higher resolution. This is known as SNP ascertainment bias [32], and implies that diversity which has accumulated in the local *S.* Typhi H58 population of Kolkata in the last decade or so is being collapsed into just a few haplotypes using our SNP typing method. Despite this, the fact that both major H58 lineages and the ancestral node were detected at high frequency in Kolkata indicates that H58 *S.* Typhi has been present in this location for some decades. This is similar to the pattern observed in Kenya where both H58 lineages have been observed at high frequency [21], but quite unlike Vietnam or Nepal where lineage I or II dominated, respectively.

### Antimicrobial resistance

In this study, as in others, antimicrobial resistance was frequent among H58 *S.* Typhi. All MDR isolates were from the H58 group, similar to recent observations in Vietnam [20], Kenya [21] and global collections [33]. Nal resistance was frequent among all H58 subtypes except H58-B (25% of H58-B; 95% of all other H58, see Table 2), although it was also frequent among common non-H58 haplotypes H42 (46%) and H14 (76%). Interestingly, *S.* Typhi H42 was also common in the Nepal study (19% of *S.* Typhi), yet Nal resistance in that location was observed only among H58-G isolates. Taken together, these observations suggest that while MDR is now largely restricted to H58 *S.* Typhi, Nal resistance arises frequently in *S.* Typhi of a diverse range of haplotypes.

### Household clustering of typhoid

Several households experienced more than one typhoid infection during the study. Among multiple cases occurring in the same household within a 2-month period, nearly all (10/12) were caused by identical infecting *S.* Typhi haplotypes, consistent with intra-household transmission or a common source (Table 4). In the post-vaccination period, eight such putative transmission events were detected in control clusters (assigned to Hepatitis A vaccine) and none were observed in Vi vaccine clusters, possibly reflecting protection via vaccination and/or herd immunity in these clusters. Three cases of relapse were identified (two infections with the same haplotype in a single individual). Although each pair of relapse isolates had an identical haplotype the haplotype was different in each individual, although all exhibited some form of antimicrobial resistance (Nal^R^ and/or MDR), suggesting that relapse may be associated with antimicrobial failure.

### Impact of the Vi vaccine

In addition to providing a snapshot of the *S.* Typhi population circulating in a localized region of Kolkata, this study offers the first insight into the impact of the introduction of Vi typhoid vaccine upon a local *S.* Typhi population. Our data indicate the incidence of all haplotypes of *S.* Typhi was similarly reduced among Vi vaccinated individuals (Table 1, Figure 2). *S.* Typhi isolated from Vi vaccinated individuals included several distinct haplotypes, which could be further differentiated by antimicrobial resistance phenotypes (Table 3). All *S.* Typhi isolates expressed Vi during laboratory culture. Thus, it is likely that ‘breakthrough’ cases of typhoid fever among vaccinees is due to subtle variations in the regulation of Vi expression *in vivo* and/or to host factors, and not to lineage-associated differences in Vi expression.

### Conclusions

The *S.* Typhi population responsible for typhoid fever in Kolkata is genetically and phenotypically diverse, displaying a wide range of haplotypes and antimicrobial susceptibility phenotypes. However the H58 haplotype dominates, and is responsible for the majority of MDR and quinolone resistant *S.* Typhi infections. The Vi polysaccharide vaccine was effective against infections with all *S.* Typhi haplotypes.

## Supporting Information

Table S1 ***S.*** **Typhi SNP loci assayed in this study.** SNPs are identified by their coordinate within the *S.* Typhi CT18 reference genome (NC_003198).
(XLS)
